# Targeting Myeloid-Derived Suppressor Cells for Premetastatic Niche Disruption After Tumor Resection

**DOI:** 10.1245/s10434-020-09371-z

**Published:** 2020-11-30

**Authors:** Fan Tang, Yan Tie, Weiqi Hong, Yuquan Wei, Chongqi Tu, Xiawei Wei

**Affiliations:** 1 Laboratory of Aging Research and Cancer Drug Target, State Key Laboratory of Biotherapy, National Clinical Research Center for Geriatrics, West China Hospital, Sichuan University, Chengdu, Sichuan People’s Republic of China; 2 Department of Orthopeadics, West China Hospital, Sichuan University, Chengdu, Sichuan People’s Republic of China; 3 Department of Oncology, Sichuan Cancer Hospital and Institute, Sichuan Cancer Center, School of Medicine, University of Electronic Science and Technology of China, Chengdu, Sichuan People’s Republic of China

## Abstract

Surgical resection is a common therapeutic option for primary solid tumors. However, high cancer recurrence and metastatic rates after resection are the main cause of cancer related mortalities. This implies the existence of a “fertile soil” following surgery that facilitates colonization by circulating cancer cells. Myeloid-derived suppressor cells (MDSCs) are essential for premetastatic niche formation, and may persist in distant organs for up to 2 weeks after surgery. These postsurgical persistent lung MDSCs exhibit stronger immunosuppression compared with presurgical MDSCs, suggesting that surgery enhances MDSC function. Surgical stress and trauma trigger the secretion of systemic inflammatory cytokines, which enhance MDSC mobilization and proliferation. Additionally, damage associated molecular patterns (DAMPs) directly activate MDSCs through pattern recognition receptor-mediated signals. Surgery also increases vascular permeability, induces an increase in lysyl oxidase and extracellular matrix remodeling in lungs, that enhances MDSC mobilization. Postsurgical therapies that inhibit the induction of premetastatic niches by MDSCs promote the long-term survival of patients. Cyclooxygenase-2 inhibitors and β-blockade, or their combination, may minimize the impact of surgical stress on MDSCs. Anti-DAMPs and associated inflammatory signaling inhibitors also are potential therapies. Existing therapies under tumor-bearing conditions, such as MDSCs depletion with low-dose chemotherapy or tyrosine kinase inhibitors, MDSCs differentiation using all-trans retinoic acid, and STAT3 inhibition merit clinical evaluation during the perioperative period. In addition, combining low-dose epigenetic drugs with chemokine receptors, reversing immunosuppression through the *Enhanced Recovery After Surgery* protocol, repairing vascular leakage, or inhibiting extracellular matrix remodeling also may enhance the long-term survival of curative resection patients.

Surgical resection is a common therapeutic option for malignant solid tumors. However, recurrence and metastatic rates after surgery are relatively high.^1^,^2^ It has been hypothesized that premetastatic niches develop in distant organs after surgery, providing “fertile soils” for circulating tumor cells (“seeds”) colonization.^3^,^4^ Recent studies have documented that after surgery, myeloid-derived suppressor cells (MDSCs) are the key immunosuppressive cells and contribute to the postoperative development of premetastatic niches at distant sites.^5^ Therapies aimed at disrupting such niches may offer long-term benefits for solid tumor patients.^6^ However, the mechanisms involved in the postoperative premetastatic niche development have not been established, and potential therapies are still in development. Phenotyping the MDSCs recruited to distant organs after surgery and understanding the associated mechanisms may significantly inform therapeutic development. In this study, we review the roles of MDSCs in premetastatic niche formation after surgery and highlight potential anti-MDSCs therapies designed to disrupt this process. Such therapies may enhance overall survival after primary tumor resection.

## MDSCs and Their Roles in Premetastatic Niche Formation

MDSCs are immature myeloid cells that exhibit immunosuppressive functions. Two categories of MDSCs have been described: granulocytic-MDSCs (G-MDSCs) and monocytic-MDSCs (M-MDSCs). In mice, MDSCs are defined as the co-expression of CD11b and Gr-1, which belong to myeloid lineage differentiation antigens. Gr-1 is composed of two different epitopes, Ly6G and Ly6C.^7^ Specifically, M-MDSCs are CD11b^+^ Ly6G^−^ Ly6C^high^, while G-MDSCs are CD11b^+^ Ly6G^+^ Ly6C^low^.^7^ In humans, the definitive identification of MDSCs is challenging. Generally, M-MDSCs are CD14^+^ HLA-DR^−/low^, whereas G-MDSCs are CD11b^+^ CD15^+^ CD14^−^ CD33^+/low^ CD66b^+^.^8^ M-MDSCs are predominantly derived from peripheral blood monocytes, whereas G-MDSCs are derived from the aberrant myelopoiesis and alternative activation of mature neutrophils from peripheral blood monocytes.^8^ Early-stage MDSCs (e-MDSC), also proposed for lineage-negative (Lin^−^) HLA-DR^−^CD33^+^ cells, have been identified in humans.^9^ e-MDSCs are comprised of more immature progenitors and exhibit immune suppressive functions through Arg-1-expressing.^10^ Both M-MDSCs and G-MDSCs are present in distant organs and contribute to premetastatic niche formation after surgery.^11^,^12^

A premetastatic niche is a premetastatic microenvironment in distant organs that is characterized by immunosuppression, inflammation, angiogenesis, and vascular permeability, organotropism, lymph-angiogenesis, as well as reprogramming.^13^ Primary cancers induce premetastatic niches in the lungs and liver.^14^,^15^ MDSCs promote this induction process through their immunosuppressive, inflammatory, and angiogenic functions.^16^ Cytokine secretion by MDSCs alters T cell and NK-cell functions, creating a locally immunosuppressive environment. G-MDSCs suppress T-cell responses by producing reactive oxygen species (ROS) through an antigen-specific approach.^17^ M-MDSCs secrete large amounts of nitric oxide (NO), Arg-1 and immunosuppressive factors, such as TGF-β and IL-10 that impair antigen-specific and non-antigen specific T-cell responses.^18^ In addition, MDSC-secreted cytokines, such as MMP9, VEGF, and Bv8 promote neovascularization. MDSCs enhance inflammation by secreting proinflammatory cytokines or by chemoattracting other immune cells, such as macrophages.^16^ Given the critical role of MDSCs in premetastatic niche development, characterizing changes in MDSC numbers and function after surgery may reveal novel therapeutic approaches for postoperative metastasis.

## MDSCs Persist in Distant Organs After Primary Tumor Resection

**Fig. 1:**
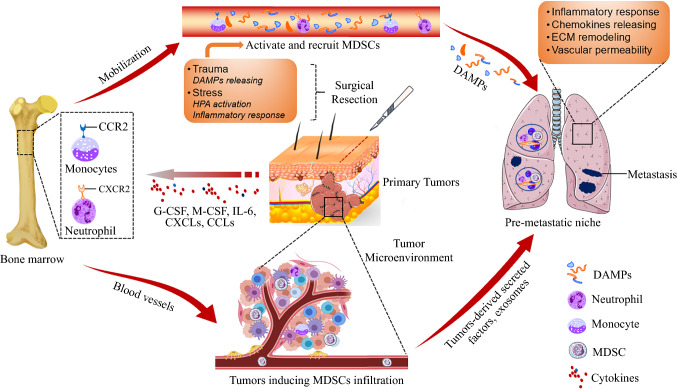
Surgical resection and postoperative premetastatic niche. Under tumor-bearing conditions, primary tumors secrete cytokines, including growth factors, chemokines that mobilize and recruit MDSCs into the tumor microenvironment. During the progression of primary tumors, tumor-derived secretory factors and exosomes mediate the movement of MDSCs into distant organs for premetastatic niche formation. After tumor resection, the circulating MDSC levels are decreased in some tumors. However, the 2-week period after surgery is accompanied by stress responses and immune dysfunctions with large cytokine level alterations. During the 2-week period after surgery, persistent MDSCs with enhanced immunosuppression by surgical stress and trauma in distant organs, such as the lung or liver are critical for premetastatic niche formation, which determine the long-term survival of patients subjected to local tumor resection

The circulating MDSC pool expands with cancer progression.^19^ Primary tumors secrete factors and exosomes that direct MDSCs to distant sites, thereby elevating MDSC numbers at these sites before surgical resection. In some solid tumors, presurgery circulating MDSC levels correlate with cancer recurrence rates after tumor resection.^20^,^21^ Although tumor resection suppresses tumor burden, thereby suppressing the amounts of circulating MDSCs, it does not deplete MDSCs fully.^22^ Preclinical studies have reported that significant numbers of functional, immunosuppressive G-MDSCs persist in the lungs for up to 2 weeks after tumor resection (Fig. 1).^5^,^23^ Animal model studies have shown that surgically stressing mice increases the number of splenic MDSCs by approximately 2.5-fold relative to controls, possibly through NK-cells impairment.^24^ Clinically, postsurgical elevation of circulating MDSCs has been observed in multiple solid tumors (Table 1).^25^–^31^ In addition, noncancer-related surgical procedures, such as cardiac bypasses and hip replacements, are associated with MDSCs expansion.^32^,^33^ Patients exhibiting elevated MDSCs show extensive surgical tissue damage, suggesting that surgical trauma elevates MDSCs. Furthermore, primary tumor resection enhances the immunosuppressive functions of MDSCs. For example, in an allograft tumor model, CD11b^+^ CD33^+^ HLA-DR^−^ MDSCs isolated 24 h after lung cancer surgery were found to be more potent at inducing angiogenesis and tumor growth compared with presurgery MDSCs.^25^ After tumor resection, infiltrating MDSCs induce epithelial-to-mesenchymal transition and metastases by upregulating TGF-β, VEGF, and IL-10 signaling.^34^ Postsurgical MDSC expansion is associated with impaired cellular immunity that increases metastatic risk.^35^ In colorectal cancer, surgical stress impairs IFN-γ secretion by NK cells, possibly due to the expansion of CD14^+^ monocytic cells (M-MDSCs).^11^ Colorectal cancer surgery expands the G-MDSCs pool, which in turn elevate scavenger receptor expression by NK cells. Moreover, postoperative lipid accumulation due to scavenger receptor expression by NK cells is associated with increased postoperative metastases.^12^ Mouse models have revealed that surgical stress enhances the MDSC numbers while suppressing the number of tumor-associated, antigen-specific, CD8^+^ T cells that produce IFN-γ.^36^ Therefore, both G-MDSCs and M-MDSCs are essential in the establishment of premetastatic microenvironments after primary tumor resection. A comprehensive, postsurgical characterization of MDSCs and elucidation of their underlying mechanisms may reveal novel strategies for improving postoperative cancer outcomes.

**Table 1:** Clinical evidence confirming the elevation and enhanced immunosuppressive function of MDSCs after surgery

Disease	Surgery	MDSCs subtype	Biological function of MDSCs	Reference
Lung cancer	Thoracotomy	CD11b^+^CD33^+^HLA-DR^–^ MDSCs	More efficient in promoting angiogenesis and tumor growth than MDSCs isolated before surgical operation	25
Lung cancer	Thoracotomy	CD11b^+^CD33^+^HLA-DR^–^CD14^+^MDSCs	Surgery-induced M-MDSCs were more efficient in suppressing T-cell proliferation, more potent in expending Treg when cocultured with autologous T cells in vitro	26
Bladder cancer	Radical cystectomy	Lin^−^ CD11b^+^ CD33^+^ MDSCs	Every unit increase in MDSC count from surgery to 2 day postoperatively, the odds of infection rate 90 day after surgery increased by 2.5%	27
Gastric cancer	Resection surgery	CD14^+^ HLA-DR^−^ CD11b^+^ CD33^+^ MDSCs	Produced IDO and arginase and suppressed T cell functions	28
Prostate cancer	Radical prostatectomy	CD14^−^HLA-DR^−^CD33^+^CD11b^+^ MDSCs	–	29
Breast Cancer	Radical or partial mastectomy	CD33^+^HLA-DR^−^ CD15^+^CD11b^+^ MDSCs	Increased levels of MDSC levels were associated with decreases in the number of NK cells	30
Esophageal cancer	Radical resection	G-MDSC	A high level of G-MDSCs can be used to determine the incidence of sepsis in preoperative esophageal cancer patients	31
Non-cancer disease	Hip arthroplasty	CD11b^+^CD14^+^CD33^+^HLA-DR^low^ MDSCs	Surgery evoked a coordinated functional response that was restricted to 6 major intracellular signaling molecules (STAT1, STAT 3, STAT 5, p38, S6, CREB)	32
	Cardiopulmonary Bypass	CD15^+^CD11b^+^CD14^−^HLA-DR^−^MDSCs	MDSCs expansion was related to the impairment of T cell proliferation, cytotoxicity, and IFN-γ secretion	33

## Mechanisms of MDSCs Infiltration After Primary Tumor Resection

**Fig. 2:**
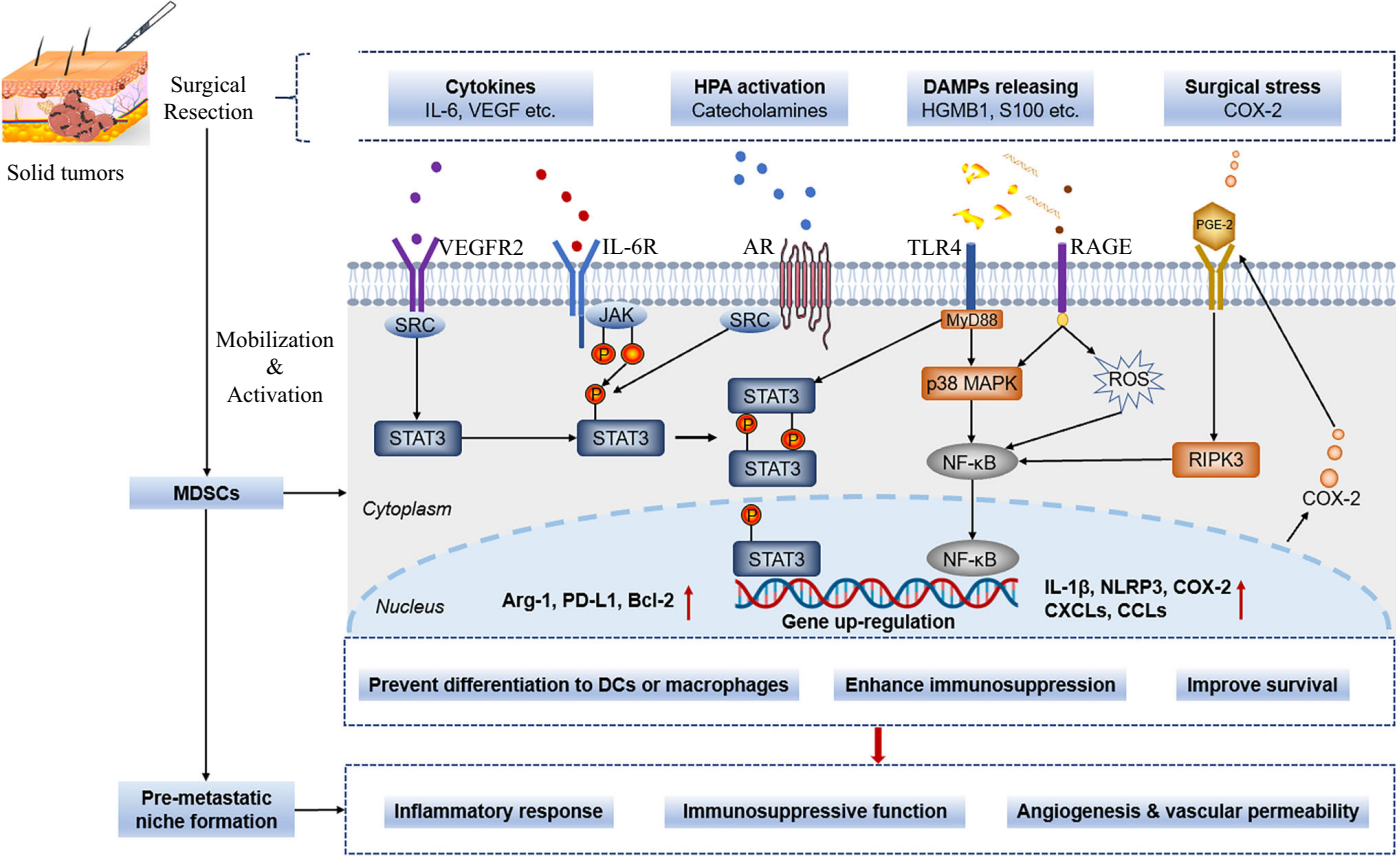
Molecular mechanisms of surgical procedures on mobilizing and enhancing functions of MDSCs. Surgical stress activates HPA axis, leading to catecholamine secretion. The catecholamines interact with AR on MDSCs that activate STAT3. In addition, high amounts of cytokines, such as IL-6, VEGF et al. activate STAT3 within MDSCs. Besides, DAMPs released after surgical trauma are recognized by PPR, especially TLR4 or RAGE that activate NF-κB. Secretion of COX-2 enhances PGE-2 synthesis, which activates NF-κB by upregulating RIPK3. Activation of the two key transcription factors (STAT3 and NF-κB) lead to the escalation of a downstream cascade. Highly activated STAT3 prevent MDSCs from rapidly differentiating into dendritic cells and macrophages. The elevated COX-2, secreted by MDSCs, lead to a positive feedback with PGE-2 and RIPK3 signal. Moreover, elevated Arg-1 and PD-L1 expression after STAT3 and NF-κB activation enhances the immunosuppressive function of MDSCs. Finally, the expression of anti-apoptotic gene (Bcl-2) improves the survival of MDSCs

The mechanisms underlying MDSCs persistence in distant organs after surgery have not been fully established. After surgery, the expansion, recruitment, and differentiation of MDSCs is regulated by various factors, such as cytokines and chemokines. Anesthetics and hormones, including catecholamines, glucocorticoids, and prostaglandin secretion in response to surgical stress, promote MDSC expansion.^37^–^40^ In addition, surgery may lead to unavoidable soft tissue damage and release of damage-associated molecular patterns (DAMPs) into circulation. In turn, DAMPs trigger local and systemic inflammatory responses, promoting MDSCs mobilization. Furthermore, some DAMPs may directly recruit and activate MDSCs, thereby enhancing their immunosuppressive functions. In the following section, we discuss the impacts of surgery on MDSC numbers and functions (Fig. 2).

## Surgical Stress Promotes MDSCs Expansion

Tumor resection is associated with relative degrees of physical, physiological, and psychological stress. Surgical stresses, as well as anesthesia, are associated with MDSC expansion. Dexmedetomidine, an α2-adrenoceptor agonist induces postoperative M-MDSC proliferation in lung cancer patients. Dexmedetomidine-induced MDSC expansion promotes tumor metastasis by enhancing VEGF expression.^37^ Surgical stress activates the hypothalamic–pituitary–adrenal (HPA) axis, elevating catecholamines and glucocorticoid levels. Because MDSCs express the receptors for these hormones, their functions may be altered by exposure to them. Catecholamine interactions with adrenergic receptors (AR) on MDSCs activate β2-AR signaling that enhances MDSCs proliferation. By proliferating, their immunosuppressive functions also are elevated. β-AR antagonists suppress MDSCs proliferation.^38^ In vitro studies have shown that MDSCs accumulation after host trauma is induced by glucocorticoids. For example, a murine trauma model revealed that endogenous glucocorticoids promote MDSC expansion.^39^ Furthermore, glucocorticoid receptor signaling suppresses HIF-1α and HIF-1α-dependent glycolysis in MDSCs, promoting their immunosuppressive activities.^41^ Prostaglandins (PG) play key roles in inflammatory responses, and their levels are significantly elevated in inflamed tissues or surgical sites. PG induces MDSCs and their suppressive functions. PG biosynthesis from arachidonate is modulated by cyclooxygenase (COX) isoenzymes and is blocked by nonsteroidal anti-inflammatory drugs, including selective COX-2 inhibitors.^40^

## Surgical Trauma Releases DAMPs to Activate, Expand, and Recruit MDSCs

Surgical trauma is accompanied by inevitable soft tissue damage and release of numerous internal signals (DAMPs) into circulation. These internal danger signals induce innate immune responses and systemic inflammation. Some DAMPs function as direct chemoattractants and recruit MDSCs. For example, S100A8/A9 (also calprotectin or MRP8/14) is a heterocomplex of 2 S100 calcium binding proteins. These S100 alarmins released after surgery are MDSC potent chemoattractants.^42^ In addition, S100 activates serum amyloid A3, which directly recruits MDSCs into the lungs, facilitating metastasis.^43^ High mobility group box 1 (HMGB1) is an evolutionarily ancient nuclear protein that regulates various intracellular and extracellular processes. HMGB1 secretion after tissue damage modulates signaling pathways that attenuate T-lymphocyte responses and enhance MDSC expansion.^44^ In colon cancer, elevated HMGB1 levels released into the peritoneal cavity recruit vast amounts of MDSCs, promoting peritoneal metastasis after curative surgery.^45^ HSP70, another important DAMP, effectively suppresses G-MDSC and M-MDSC populations.^46^ In addition, DAMPs extracellular ATP enhances MDSC dysfunction through the ATP receptor (P2 × 7R) and NLRP3 inflammasome activation.^47^ Complement factors also are secreted during surgical trauma. Studies in mouse models have shown that C5a receptors, C5a-R1, and C5a-R2 elevate MDSC levels in melanoma. C5aR1 knockout suppressed the in vivo levels of MDSCs, macrophages, and regulatory T lymphocytes.^48^

When DAMPs reach distant organs, such as lungs, their clearance by host immune cells is followed by the activation of inflammatory or pyroptosis signals. During this process, the secretion of the NLRP3 inflammasome, NF-κB transcription, and IL-1β is upregulated. IL-1β enhances the generation, accumulation, and activity of MDSCs by activating IL-1β-inducible growth factors, cytokines, CXCR2-directed chemokines, and adhesion molecules that are associated with G-MDSC infiltration.^49^ Specifically, the activation of the NLRP3 inflammasome, NF-κB and IL-1β elevates chemokine secretion, including CXCLs or CCLs that recruit MDSCs into the premetastatic niches.^50^

DAMPs recognition by pattern recognition receptors (PRRs) on MDSCs triggers a cascade of events that involve NF-κB and STAT3. These events activate MDSCs and enhances MDSC-mediated cell migration and immunosuppression (Fig. 2). For instance, the downstream mediators of DAMP signaling, such as prostaglandin E2 (PGE-2) and IL-10, induce immunosuppressive MDSCs. In a mouse model of mammary carcinoma, S100A8/A9 bound to a receptor for advanced glycation end products (RAGE) on MDSCs, promoting their migration and accumulation through NF-kB signaling.^42^ STAT3 upregulates the S100A8/9 proinflammatory proteins, which enhances MDSCs accumulation in a feedback loop. Elevated S100A8/9 levels suppress myeloid progenitor cell differentiation. HMGB1 enhances MDSC viability by driving them into a proautophagic state.^51^ In addition, MDSCs recruited into the pre-metastatic lungs through the C5a/C5a R1 axis modulate premetastatic angiogenesis.^52^

## Surgery-Induced Cytokines Enhance MDSC Mobilization and Function

Surgical stress and trauma activates cytokines that initiate MDSC mobilization and their immunosuppressive activities. These cytokines, including interleukins, growth factors, colony-stimulating factor (CSF), and chemokines, are immediately elevated after surgery. They promote MDSC generation, chemotaxis, and activity (Table 2).^53^–^85^ TNF-α expression is elevated soon after surgical trauma. It activates an inflammatory cascade. TNF-R2 signaling promotes MDSC survival and peripheral accumulation by upregulating cellular FLICE-inhibitory protein (c-FLIP) and suppressing caspase-8 activity.^70^ Proinflammatory cytokine levels, including IL-6, G-CSF, and CCL2, are elevated within hours after surgery.^86^ In the lungs, IL-6 interacts with IL-6R on MDSCs to activate STAT3 transcriptional activity, thereby promoting premetastatic niche formation and immunosuppression.^54^ STAT3 activation by IL-6 and other cytokines, such as IL-10, hepatocyte growth factor, and CSF, enhances Arg-1 and PD-L1 expression by MDSCs. STAT3 activation also elevates the expression of Bcl-2 that enhances MDSCs survival. Surgical trauma elevates G-CSF and GM-CSF levels, thereby inducing MDSCs. G-CSF treatment enhances MDSCs infiltration and elevates the expression of immunosuppressive factors, including Arg-1, IL-10, and ROS.^60^ Circulating levels of C-reactive protein (CRP), which is expressed in the liver, are significantly elevated during inflammation. CRP serum levels rapidly rise from approximately ≤ 3 µg/ml to > 500 µg/ml in response to proinflammatory cytokine stimulation. CRP has been shown enhance MDSC numbers and activity in vivo.^87^ In a cancer mouse model, VEGF was shown to promote the accumulation of Gr1^+^CD11b^+^ cells by interacting with VEGF-R2 and activating JAK2 and STAT3.^88^ PGE-2 modulates the in vivo development and activities of MDSCs.^89^,^90^ It enhances the nuclear accumulation of NF-κB in M-MDSC that divert their response to IFN-γ towards NO-mediated immunosuppression.^91^

**Table 2:** Cytokines released after surgery promoted the mobilization and enhanced the function of MDSCs

Cytokines	Name	Biological function in MDSCs	Biological function in surgery
Interleukins	IL-6	IL-6 potentially expands peripheral MDSCs;^53^ major cytokine among IL-6 family cytokines responsible for STAT3 activation and premetastatic niche^54^	Main proinflammatory cytokine responds to surgery and the magnitude of IL-6 elevation correlates with the extent of surgical trauma severity; the elevation of IL-6 associated with postoperative adverse outcome^55^,^84^
	IL-8	Attracts MDSCs and elicits extrusion of neutrophil extracellular traps^56^	Proinflammatory cytokine responds to surgical stress^57^
Colony-stimulating factors (CSF)	GM-CSF	Recruit and expand MDSCs, promote migration and differentiation of MDSCs^58^	GM-CSF ameliorates microvascular barrier integrity via pericyte-derived Ang-1 during wound healing^59^
	G-CSF	Mobilize G-MDSCs to the lung pre-metastatic niche^60^	Angiogenic circulating factor responding to surgery^61^
Chemokines	CXCL1	Recruits CXCR2-positive MDSCs to form a premetastatic niche, promoting liver metastases^62^	Proangiogenic chemokine, that participate wound healing
	CCL2	CCL2/CCR2 axis is important for MDSC recruitment^63^	Evaluation of CCL2 help guide postsurgical management for clear-cell renal cell carcinoma patients^64^
	SDF-1/CXCL 12	SDF-1/CXCR4-mediated recruitment of MDSCs from bone marrow^65^	Chemokine, involving wound healing^66^
Growth factors	VEGF	Activate NF-κB signals, producing CXCL1 to recruit CXCR2^+^ MDSC^62^	One of the most potent proangiogenic factors during wounds healing^67^
Interferon	IFN-γ	IFN-γ significantly upregulated iNOS expression in M-MDSCs^68^	Mediating postoperative proinflammatory responses^69^
Tumor necrosis factor (TNF)	TNF-α	Cytokine attracting neutrophils and monocytes to pre-metastatic niche; Signaling of TNF-R2 promoted MDSC survival through upregulation of c-FLIP and inhibition of caspase-8 activity^70^	First cytokine responding to injury, trigger an inflammatory cascade. Endogenous wound TNF-α down-regulates collagen synthesis during normal wound healing^71^
Transforming growth factor (TGF)	TGF-β	Factor secreted by MDSCs, that with strong immunosuppressive function^166^	TGF-β exhibits two postoperative peaks of secretion at 2 h and 3–4 days. Stimulates angiogenesis and fibroblast proliferation^72^
Extracellular matrix	MMP9	MMP-9-cleaved OPN fragment, OPN-32 kDa, was responsible for MDSCs expansion^73^	Serum MMP-9 increased significantly 4 days after surgery and was still high 30 days after surgery; play a role in normal tissue remodeling events^74^
	LOX	Promote the ECM remodeling to recruit MDSC^75^	A key enzyme required for crosslinking and deposition of insoluble collagen, and targeting LOX might be an approach to reduce adhesions^76^
	Fibronectin	Fibronectin is a large glycoprotein capable of interacting with various ECM molecules produced by a variety of cell types and involved in cell attachment and chemotaxis^79^	ECM derived DAMPs after surgery that activate inflammation and monocyte activation; also participated in wound healing^80^
DAMPs	S100A8/9	S100A8/A9 imaging reflected MDSC abundance and the establishment of an immunosuppressive environment in premetastatic lung tissue^77^,^78^	Causing neutrophil migration to inflammatory sites; a biomarker of postoperative organ injury^85^
Prostaglandin-endoperoxide synthase	COX-2	Catalyzed the synthesis of PGE-2, which exacerbated the immunosuppressive activity of MDSC^81^	An enzyme responsible for the production of PGs that respond to surgical stress^82^

Surgically activated chemokines recruit MDSCs to distant organs. They also induce MDSCs development in the bone marrow and maintenance of their immunosuppressive activity.^92^ In colon cancer, surgical trauma promotes tumor progression by downregulating CXCL4 while promoting MDSC recruitment.^93^ IL-8, a proinflammatory cytokine, and main CXCR2 ligand, is elevated within hours after surgery. It chemotactically recruits MDSC by interacting with CXCR2.^56^ In cancer patients, CXCR2 chemokine receptor agonists lead to the extrusion of neutrophil extracellular traps (NETs) by neutrophils, and G-MDSCs.^94^ The CCL2/CCR2 signaling axis recruits M-MDSCs to distant organs. Under surgical stress, CCL2 levels rise and remain high for approximately 2 months after surgery.^95^

## Surgery Promotes Extracellular Matrix Remodeling

The immediate effects of tumor resection are associated with extracellular matrix (ECM) remodeling, causing the lungs to be more vulnerable to metastasis. ECM remodeling is mediated by lysyl oxidase (LOX), a copper-dependent amine oxidase that catalyzes an important enzymatic step in collagen crosslinking.^96^ In the premetastatic niche, LOX-mediated collagen IV crosslinking in the basement membrane is essential for MDSCs recruitment.^97^ In response to surgery, elevated LOX plasma levels promote lung ECM remodeling, raising the risk of tumor cell seeding during the immediate post-operative period. Blocking the activity of LOX in peripheral blood suppresses tumor cell seeding in lungs. This study emphasizes the essential role of ECM remodeling in promoting tumor cell seeding after surgery; there are no changes in CD11b^+^ cells colonizing the premetastatic sites, as well as pulmonary permeability.^75^ Solid tumor surgeries elevate plasma LOX levels, thereby promoting lung ECM remodeling. Therapeutically targeting the LOX family members may prevent metastases and cancer progression after tumor resection.

## Surgery Enhances MDSC Infiltration by Promoting Vascular Permeability

Surgically induced local inflammation and neutrophil infiltration in the lungs is associated with elevated vascular permeability.^98^ In turn, higher pulmonary vascular permeability promotes MDSCs infiltration, which promotes vascular permeability by secreting MMP9 into the premetastatic lungs.^99^ Therefore, increased vascular permeability is a key step in MDSCs settlement. Cyclic GMP-AMP synthase (cGAS) is a DNA sensor that can be activated by endogenous DNA, including extranuclear chromatin and mitochondrial DNA. Activation of cGAS signaling leads to an innate immune response that preserves organismal homeostasis.^100^ Transcriptional regulators Yes-associated protein (YAP) and its paralog TAZ are the major downstream proteins of the Hippo signaling pathway, which is critical for endothelial homeostasis.^101^ Release of large amounts of circulating mitochondrial DNA due to surgical trauma activates cGAS signaling and suppresses YAP-mediated endothelial cell proliferation. These effects promote vascular inflammation.^102^ This process enhances vascular leakage and, therefore, MDSCs mobilization to distant organs. Surgical trauma-induced proinflammatory cytokines, such as IL-6, mediate pulmonary vascular permeability in a two-hit model of ventilator-associated lung injury.^103^ Anti-inflammation suppresses proinflammatory cytokine secretion and maintains vessel permeability.^104^ Surgical activation of the complement system triggers a cascade of events that promote vascular permeability.^105^ In this process, neutrophils, recruited by C5a, secrete ROS, PG and leukotriene.^106^ Therapeutic options that repair leaking vessels in the premetastatic niche have been shown to prevent the outgrowth of circulating cancer cells while sensitizing lung metastases to immunotherapy.^107^

## Therapies Targeting MDSCs for Premetastatic Niche Disruption After Surgery

Potential therapeutic strategies against persistent MDSCs in distant organs include direct anti-MDSC therapies and therapies aimed at minimizing the impact of surgery on MDSCs. Depleting MDSCs using low-dose chemotherapy or tyrosine kinase inhibitors suppresses MDSC chemotaxis and proliferation, while reversing the immunosuppressive functions of MDSCs (Table 3). Additionally, anti-postoperative MDSC therapy should be considered, because the perioperative period is associated with a heightened risk of complications, including infections and slow wound healing due to immunosuppression.

**Table 3:** Possible and rational therapies targeting MDSCs for pre-metastatic niche disruption after tumor resection

Strategies	Drugs	Clinical trial	Mechanisms	Reference
Anti-surgical stress	β-blockade (Propranolol)	NCT03152786/NCT02013492	Block the β2-AR signal in MDSCs	109
	COX-2 inhibitor (Celecoxib, Etodolac)	NCT03896113/NCT03864575/NCT03838029	Disruption of the COX-2/PGE-2 signals; reducing MDSC recruitment and differentiation, repressing MDSC-associated suppressive factors such as Arg-1 and ROS production	81
Anti-DAMPs	Anti-HMGB 1 antibody	Preclinical	Ameliorated the trauma-induced attenuated T-cell responses and accumulation of MDSCs in the spleens seen 2 days after injury	44
	S100A8/A9 neutralizing antibody	Preclinical	Neutralizing extracellular S100A8/A9	119
Anti-inflammatory signals	NF-κB inhibitor (BAY11-7082)	Preclinical	Inhibiting NF-κB mediated CXCL1, CXCL2 and CXCL5 production, that reduce the chemotaxis of MDSCs; Inhibition NLRP3 inflammasome activation and the increased pyroptosis	124
	NLRP3 inhibitor (MCC950/CY-09/OLT1177/Tranilast/Oridonin)	Preclinical	Inhibition NLRP3 inflammasome activation and the following IL-1β secretion	121
	IL-1β (Anakinra)	NCT02780583/NCT04359784	Inhibit the IL-1β inducing CCL5, CXCL12, CCL2, and CXCL5 expression	50
Epigenetic drugs	HDAC (Entinostat)	NCT01207726/NCT03501381/NCT03552380	Downregulating CCR2 and CXCR2 expression; promoting MDSC differentiation into a more-interstitial macrophage-like phenotype	5
	DMNT (5-azacytidine)	NCT01207726/NCT03709550		
Depletion of MDSCs	Chemotherapy (Gemcitabine/5-FU)	NCT04331626/NCT01803152/NCT02090101	Depletes G-MDSCs, M-MDSCs, macrophages, and eosinophils	142
	TKI(Pazopanib/Bevacizumab)	NCT01832259/NCT01218048	Blockade of VEGF and c-KIT signaling; inhibit STAT3	129
Differentiating MDSCs	ARTA	NCT02403778	Inhibits retinoic signaling to shift the differentiation of MDSC into mature myeloid cells, such as macrophages and dendritic cells	147
Blocking MDSCs trafficking	CXCR2 inhibitor (AZD5069, Reparixin, SX-682)	NCT03161431/NCT02499328/NCT02370238	Inhibit CXCR2 mediated MDSCs trafficking	94
	CCR2 inhibitor (BMS-813160, RS504393)	NCT04123379/NCT03767582/NCT03496662	Improved the prognosis by blocking chemotaxis of M-MDSCs	142
Inhibit MDSCs activity	Stat3 inhibitor (AZD9150)	NCT02499328	A marked decrease in G-MDSC within the peripheral blood mononuclear cells	158
	NF-κB inhibitor (BAY11-7082)	Pre-clinical	Inhibit the activated NF-kB signal induced by DAMPs-PRR interaction	42
Targeting the ECM remodeling	LOX inhibitor (β-aminopropionitrile)	Pre-clinical	Reduce the surgery induced hypoxia related LOX, and ECM remodeling	161
Repair vascular leakage	IL-6 inhibitor (Tocilizumab)	NCT04370834)	Inhibit IL-6 mediated inflammatory response the repair the vascular leakage	164
	C5 antibody (Ravulizumab)	NCT04369469	Inhibit the C5a-C5R signal on vascular endothelial cells	165
Restore the immunity after surgery	TGF-β inhibitor (LY3200882/LY2157299/EW-7197)	NCT04031872/NCT03143985/NCT02452008	Reverse the MDSC secreted TGF-β mediated immunosuppression	166
	PDE5 inhibitor (Sildenafil, Tadalafil)	NCT02544880	Targeting MDSC expression and function of Arg-1 and iNOS	167–169
	ERAS	NCT02644603	Pre-rehabilitation, nutrition, pain management to rapid restore immunity after surgery	172

## Reduced Surgical Stress Induces MDSCs Activation

*Propranolol* Short perioperative periods, days to weeks around surgery, are characterized by stress-inflammatory responses, including the secretion of catecholamines and prostaglandins.^108^ Catecholamines activate AR which are presented by all cell types, including MDSCs. Among these signals, β2-AR enhances MDSC accumulation and survival through STAT3 phosphorylation. Following STAT3 activation, MDSCs elevate the expression of pro-survival and immunosuppressive genes, such as Bcl-2 and Arg-1.^38^ Therefore, catecholamine suppression minimizes immunosuppression in the microenvironment by inhibiting MDSC recruitment.^109^ Propranolol, a pan β-AR blocker, significantly slows tumor growth in a β2-AR dependent manner. Perioperative application of propranolol disrupts premetastatic niche formation after surgery. Propranolol administration attenuates the elevation of MDSCs, indicating that after surgery, catecholamines promote MDSCs function.^110^ Ongoing clinical trials aim to elucidate the efficacy of β-blockers after tumor resection (NCT03152786) or in advanced, inoperable cancers (NCT02013492).

*COX-2 inhibitor* During trauma, COX-2 modulates immune functions by inducing PGE-2 production, which activates NF-κB signaling in a positive feedback loop that enhances COX-2 expression in MDSCs.111 Inhibition of COX-2 or PGE-2 reverses the immunosuppressive activity of MDSC and suppresses tumorigenesis.81 In mouse models, a selective COX-2 inhibitor (NS398) was shown to decrease significantly the percentage of MDSCs in the spleen and bone marrow within 48 and 72 h after traumatic stress, respectively. NS398 also inhibits arginase activity and downregulates Arg-1 expression by splenic MDSCs, thereby enhancing proliferation while inhibiting apoptosis in CD4 + T cells.112 Modulation of chemokine CXCL12 and its receptor CXCR4 by PGE-2 attracts MDSC into the tumor microenvironment. Inhibition of the COX-2 or PGE-2 receptors EP2/EP4 suppresses CXCR4 expression and MDSC responsiveness to CXCL12.113 Celecoxib, a COX-2 selective nonsteroidal anti-inflammatory drug, is used for pain and inflammation control in the perioperative period. This drug suppresses the in vitro and in vivo PGE-2 levels. Treatment of tumor-bearing mice with dietary celecoxib prevents local and systemic expansion of MDSCs.114, 115 Based the potential of the COX-2 inhibitor to regulate immune response, clinical trials are evaluating its effect (NCT03896113, NCT03864575).

Combining COX-2 inhibition and β-blockade has potential immunological and clinical benefits after cancer surgery.^116^ In breast cancer, the administration of propranolol and etodola, a PG inhibitor was shown to reduce IL-6, CRP, and IFN-γ serum levels, as well as the activity of multiple inflammation-related transcription factors, including NF-κB and STAT3.^117^ In a phase-II, randomized, clinical trial involving breast cancer patients subjected to tumor resection, the simultaneous inhibition of β-adrenergic and COX-2 pathways in the perioperative period was associated with improved long-term survival rates.^118^ A phase II, clinical trial is being performed in Israel to evaluate the efficacy of β-adrenergic blockade and COX-2 inhibition against metastasis in primary pancreatic cancer patients undergoing surgery. This trial is based on results from animal studies showing that the β-adrenergic blocker, propranolol, in combination with etodola (but neither drug alone), effectively suppressed postoperative metastasis (NCT03838029).

## Anti-DAMPs and Related Pyroptosis/Inflammatory Signaling

*Anti-DAMPs* The release of DAMPs after surgical trauma is associated with the accumulation and activation of MDSCs. Therapies that clear these intrinsic alarmins may suppress MDSC levels. Preclinical studies suggest that a monoclonal antibody that directly neutralizes DAMPs may prevent MDSCs accumulation. For example, an anti-HMGB1 monoclonal antibody was found to improve trauma-induced T-cell response attenuation and MDSC accumulation within 2 days after injury.^44^ Extracellular S100A8/A9 upregulates various inflammatory cytokines and chemokines. Kinoshita et al. developed a neutralizing anti-S100A8/A9 antibody for blocking “seed and soil” cross-talk and found that it efficiently suppressed organ tropic metastasis.^119^ Given that numerous DAMPs are released after surgery, the feasibility for the application of neutralizing antibodies is limited. Therefore, therapies targeting the downstream inflammatory signals are better options in the perioperative period.

*IL-1β blockade* IL-1β is secreted by host immune cells to promote DAMPs clearance. It has been associated with MDSC accumulation and NK-cells immunosuppression.^120^ IL-1β expressing tumor cells express higher levels of G-CSF, multiple CXC chemokines, and vascular adhesion molecules that mediate G-MDSCs infiltration and enhance angiogenic and immunosuppressive properties.^49^ Activation of MDSCs by IL-1β enhances their immunosuppressive activities through NF-κB signaling. Therefore, IL-1β inhibition may suppress MDSCs infiltration and function. Anakinra is an IL-1R antagonist that inhibits distal metastasis by blocking pyroptosis/IL-1β signaling. Anakinra suppresses the recruitment of M2-macrophages and MDSCs by inhibiting CCL2, CCL5 and CXCL5 secretion.^50^ Anakinra is under clinical trials for a certain inflammatory disease (NCT02780583) and for the control of inflammatory cytokine release (NCT04359784). Although it has not been tested in cancer, its perioperative uses may suppress immediate post-operative MDSCs accumulation in the lungs.

*NLRP3 inhibitor* The release of vast amounts of DAMPs after surgical trauma activates NLRP3 signaling in host immune cells and MDSCs. A preclinical study found that pharmacologic inhibition of NLRP3 suppressed G-MDSC tumor infiltration and significantly enhanced anti-PD-1 efficacy. The NLRP3 inflammasome was highlighted as a potential therapeutic avenue for reducing MDSCs infiltration and immunosuppression.^121^ Although some small molecules have been identified as NLPR3 inhibitors, few of them have been tested in clinical trials. These molecules include MCC950, CY-09, OLT1177, Tranilast and Oridonin, which exhibit therapeutic potentials by directly targeting NLRP3.^122^ More studies needed to determine whether these drugs inhibit postoperative MDSCs and their clinical efficacies in tumor resection patients.

*NF-κB inhibitor* NF-κB signaling is critical for the activation of MDSCs and maintaining their immunosuppressive functions. Activation of RAGE-mediated p38 MAPK and TLR4-mediated NF-κB signaling is implicated in S100A9-induced MDSC chemotaxis and activation.^123^ In addition, it is an important signal involving DAMPs-induced inflammatory responses. NF-κB activation in host immune cells promotes secretion of chemokines that recruit MDSCs. Other cytokines also mediate MDSC accumulation by relying on this signal. A preclinical study established that BAY11-7082, an NF-κB inhibitor, suppresses the secretion of CXCL1, CXCL2, and CXCL8. These chemokines mediate CXCR2^+^ MDSC recruitment.^124^ Although this drug is still under preclinical evaluation, it has exhibited a therapeutic potential in eliminating the postoperative premetastatic niche.

## Suppressing MDSC Numbers After Surgery

*Gemcitabine* Reduced-dose treatment with gemcitabine eliminates MDSC and other immunosuppressive cells. In vivo experiments in a mouse model found that despite early tumor resection, animals died of local recurrence and distant metastasis. However, adjuvant gemcitabine treatment after tumor resection prolonged survival by inhibiting MDSCs accumulation and increasing the number of NK cells at the resection margin.^125^ Treatment with gemcitabine after primary tumor resection has been shown to decrease residual G-MDSCs in the lungs and suppress metastasis.^23^ A phase IV, open, nonrandomized, single-arm, single-center study is underway. This study aims at establishing the safety and efficacy of half-dose gemcitabine in combination with anti-PD-1 antibody in non-small cell lung cancer (NCT04331626). Gemcitabine pretreatment may enhance the efficacy of dendritic cell vaccines after tumor resection, helping eliminate immunosuppressive cells. The synergistic effects of dendritic cell vaccines and gemcitabine in sarcoma are under evaluation in adults and children (NCT01803152). In this clinical trial, following tumor resection, participants undergo pheresis to isolate monocytes, which are then used to grow dendritic cells. Dendritic cell vaccination is then done 2 weeks after the third low-dose gemcitabine administration, and MDSC levels monitored.

*5-fluorouracil* Low-dose 5-fluorouracil (5-FU) was found to inhibit systemic and lung accumulation of MDSC in tumor-bearing mice.^126^ 5-FU mediated MDSC depletion was relatively strong when compared to gemcitabine and selectively triggered MDSC apoptosis in vitro and in vivo.^127^ 5-FU, alone or in combination with bevacizumab, rapidly depletes circulating MDSCs and activates caspase 1.^128^ 5-FU triggered IL-1 expression is detected in serum 24 h after treatment. A phase II clinical trial is being performed to evaluate the impact of 5-FU, bevacizumab, and Anakinra on liver metastases and vascularization in metastatic colorectal cancer (NCT02090101). However, even with low-dose chemotherapy, postsurgical immunosuppression complications including slow wound-healing and infection should be carefully considered.

*Tyrosine kinase inhibitors* VEGF is an important pro-angiogenic factor. VEGF causes immune dysfunctions by inhibiting T-cell activity, and promoting MDSC recruitment.^129^ VEGFR-1^+^ cells promote metastases by establishing premetastatic niches.^130^ By inhibiting myelopoiesis, antiangiogenic factors indirectly suppress MDSC levels. Pazopanib, a multi-tyrosine kinase inhibitor (TKI), decreases VEGFR-1^+^ MDSCs in pelvic lymph nodes, the most frequent sites of prostate cancer metastasis, disrupting premetastatic niches, and improving clinical outcomes.^131^ Presurgical treatment with cetuximab, an anti-EGFR monoclonal antibody, has undergone clinical trials in patients with locally advanced head and neck squamous cell carcinoma (NCT01218048). Patients who responded to this treatment exhibited a significant decrease in G-MDSC levels.^132^ TYRO3, AXL, and MERTK impact on the activities of MDSC and are promising targets for reversing MDSC-mediated immunosuppression in cancer.^133^ Ibrutinib, a Bruton’s tyrosine kinase and IL-2-inducible kinase, suppresses tumor development and metastasis in breast cancer by inducing MDSCs differentiation into mature dendritic cells.^134^ Bevacizumab, an anti-VEGF antibody, effectively reduces adhesion after first sternotomy by downregulating CD31, and therefore, inhibiting VEGF-induced angiogenesis.^135^ Low-dose capecitabine, in combination with bevacizumab is well tolerated and has been associated with low levels of circulating MDSCs, as well as elevated cytotoxic immune cell infiltration into the tumor microenvironment.^128^ This shows that TKIs may be effective in MDSCs management after surgery and might improve long term survival outcomes. However, more studies regarding the dosage and timing of TKI administration are needed, so as to minimize wound healing complications.

## Blocking the Recruitment of MDSCs After Surgery

*CXCR2 Blockade* The chemotaxis factor, CXCR2, is essential for G-MDSC recruitment. Anti-CXCR2 and anti-PD-1 have been shown to synergistically reduce tumor mass and enhance CD4^+^ and CD8^+^ T-cell infiltration.^136^ Three CXCR1/2 antagonists (SX-682, reparixin, and MK-7123) are being evaluated in clinical trials as adjunctive cancer therapies. SX-682 is an orally bioavailable small-molecule inhibitor of CXCR1/2 that blocks MDSC recruitment while enhancing T-cell activation. SX-682 significantly inhibits G-MDSCs trafficking without altering CXCR2 ligand expression.^137^ Preclinical studies have shown that the inhibition of G-MDSC trafficking by SX-682 enhances T-cell based immunotherapy, indicating a potential benefit against MDSC-infiltrated cancers.^138^ A study (NCT03161431) is being performed to determine whether SX-682 blocks MDSCs recruitment into tumors. Reparixin is designed to target CXCL8-CXCR1 signaling in cancer stem cells and is now in a Phase II clinical trial for triple-negative breast cancer (NCT02370238). AZD5069 is a selective small-molecule antagonist of human CXCR2 chemokine receptor with a greater than 100-fold selectivity over CXCR1 receptors.^139^ This drug is in Phase Ib/II trials for advanced solid tumors and metastatic squamous cell carcinoma (NCT02499328).

*CCR2 inhibitor* The CCL2-CCR2 chemokine axis mediates M-MDSCs recruitment into distant organs, contributing to the creation of a pre-metastatic niche.^140^ Pro-pagermanium is a CCL2 inhibitor used in Japan for chronic hepatitis B treatment. It has been shown to inhibit metastasis by suppressing premetastatic niche formation.^141^ A CCR2 antagonist, RS 504393, improved prognosis by blocking M-MDSCs chemotaxis.^142^ It has been proven that the CCR2 inhibitor alone does not improve overall survival outcomes in mice models after tumor resection. However, when combined with other drugs, including epigenetic drugs that suppress CCR2 expression by MDSCs, the treatment synergistically prolonged mice survival after tumor resection.^5^ The CCR2 inhibitor (BMS-813160) is under clinical evaluation, in combination with immunotherapy or chemotherapy against solid cancers (NCT03496662, NCT03767582, NCT04123379).

*DNA methyltransferase (DNMT) and histone deacetylase (HDAC) inhibitors* Epigenetics is an important modulator of gene expression. Epigenetic events modulate chronic inflammatory responses making them potential therapeutic targets.^143^ MDSC phenotype development has been shown to be enhanced by HDAC2-mediated epigenetic silencing of the retinoblastoma gene.^144^ In trauma patients, MDSC mediated immunosuppression develops over time, and is associated with specific epigenome modulation.^145^ Valproic acid, a HDAC inhibitor, downregulates CCR2 expression on M-MDSCs thereby suppressing M-MDSC infiltration. Valproic acid also relieves the immunosuppressive effects of MDSCs on CD8^+^ T cells and NK cells, enhancing their activation in tumors.^146^ Adjuvant epigenetic therapy using low-dose DNA methyltransferase (5-azacytidine) and histone deacetylase (Entinostat) inhibitors disrupts the premetastatic niche by downregulating CCR2 and CXCR2, thereby inhibiting MDSC recruitment and promoting their differentiation into macrophage-like cells.^5^ A combination of low-dose adjuvant epigenetic therapy and CCR2 antagonists synergistically enhance disease-free and overall survival outcomes. Most epigenetic drugs are not approved for mono-therapeutic use in solid tumors. This is because they exhibit limited clinical efficacies and severe toxicities at high doses. Therefore, using low doses that suffice to reprogram MDSCs, in combination with other anticancer agents, may provide effective treatment options, while minimizing toxicity.

## MDSCs Differentiation After Surgery

*All-trans retinoic acid (ATRA)* ATRA is a derivative of vitamin A that has been shown to strongly eliminate MDSCs in mice models and in cancer patients. ATRA enhances MDSC differentiation into mature myeloid cells by neutralizing high ROS levels.^147^ It also activates the genes responsible for cell differentiation that are less likely to favor tumor growth by differentiating MDSCs into dendritic cells, granulocytes, and monocytes.^148^ Treating a mouse sarcoma model with ATRA significantly eradicated M-MDSCs and diminished the immunosuppressive effects of G-MDSCs.^149^ ATRA administration reverted anti-VEGFR-2-induced accumulation of intratumoral MDSC, and counteracted the disorganization of tumor microvessels.^148^ Epigenetic drugs also have exhibited the potential to differentiate MDSCs.^5^ A randomized, phase II, clinical trial of advanced melanoma treatment with ipilimumab alone or in combination with ATRA is underway (NCT02403778).

## Targeting the Intrinsic Signals Responsible for MDSCs Activation

*STAT3 inhibitors* As a critical transcription factor, STAT3 promotes cell proliferation, anti-apoptosis, angiogenesis, metastasis, and immune evasion.^150^ STAT3 activation mediates MDSC survival and immunosuppressive functions. For example, M-MDSC-driven immunosuppression in pancreatic ductal carcinoma is mediated by STAT3.^151^ Galiellalactone, a STAT3 inhibitor, suppresses M-MDSC development and immunosuppression.^152^ STAT3 phosphorylation mediates the expression of Arg-1. Stattic, a STAT3-specific inhibitor restores MDSC mediated immunosuppression by rescuing Arg-1 expression after STAT3 inhibition.^153^ Downregulation of phospho-STAT3 levels in liver cancer significantly suppresses MDSC numbers and tumor burden. In addition, this process also promoted Bax-dependent apoptosis in MDSCs by upregulating pro-apoptotic genes, including Bax and cleaved caspase-3, while downregulating the anti-apoptotic factor, Bcl-2.^154^ Stimulator of interferon genes (STING) is a major sensor in host defense signaling that is considered to be a vital protein in the regulation of anti-tumor immunity and MDSC differentiation.^155^ A combination of STAT3 inhibitor and a STING agonist suppresses tumor growth, partly by reducing tumor microenvironment MDSC levels.^156^ The systemic administration of AZD9150, an antisense oligonucleotide STAT3 inhibitor, reduces G-MDSC peripheral levels in patients.^157^,^158^ AZD9150 has been trialed in combination with anti-PD-1 against advanced solid tumors and relapsed metastatic head and neck squamous cell carcinoma (NCT02499328).

## Inhibition of ECM Remodeling in Distant Organs After Surgery

LOX is a secreted copper-dependent amine oxidase that cross-links collagens and elastin in the ECM. It induces postoperative ECM remodeling in the lungs. Its expression is induced by hypoxic conditions, including surgical wounding. Postsurgical inhibitors of LOX upregulation inhibit MDSCs infiltration and postoperative metastasis. However, in the absence of a crystal structure, it is extremely difficult to design selective inhibitors.^159^ Recently, Leung et al. developed an orally bioavailable LOX inhibitor (CCT365623) with good anti-LOX potency, selectivity, pharmacokinetic properties, and anti-metastasis effects.^160^ β-aminopropionitrile (an organic compound with amine and nitrile functional groups), is an irreversible LOX inhibitor. BPAN suppresses LOX effects in vivo and in vitro.^161^ In addition, LOX expression is correlated with elevated VEGF and platelet-derived growth factor expression, and both were downregulated by β-aminopropionitrile in vivo.^162^ CCT365623, another LOX inhibitor, disrupts EGFR cell surface retention and slows in vivo primary and metastatic tumor progression.^163^ Even though these LOX inhibitors have not been introduced in clinical trials for cancer therapy, they are promising therapeutic options for premetastatic niche disruption after surgery.

## Repair of Lung Vascular Permeability After Surgery

Inflammatory cytokines promote vascular permeability in lungs, enhancing MDSCs and cancer cell infiltration. Cytokines secreted by infiltrating MDSCs promote further pulmonary vascular leakage. This effects may be inhibited by anti-inflammatory drugs, anti-proinflammatory cytokine antibodies (e.g., anti-IL-6) or the C5a inhibitor. By upregulating angiopoietin 2 and suppressing angiopoietin 1, IL-6 generates a serum profile that promotes vascular leakage.^164^ Tocilizumab is an IL-6 inhibitor used to treat the cytokine storm that is triggered by some immunotherapies, and may be effective in suppressing inflammatory responses. C5aR is upregulated in bronchial and alveolar epithelial cells, greatly contributing to lung inflammation and injury.^165^ Ravulizumab (ALXN1210) is a novel C5 inhibitor that potently inhibits C5 when administered on an extended 8-week dosing interval. A phase III, open-label, randomized, controlled study to evaluate the efficacy and safety of intravenous Ravulizumab administration relative to the best supportive care given to severe covid-19 cases, acute lung injuries, or acute respiratory distress syndrome (NCT04369469) is underway. Although these drugs have not been trialed perioperatively, they are promising agents for the repair of vascular permeability after surgery, thereby inhibiting MDSC and cancer cell infiltration.

## Inhibition of MDSC-Mediated Immunosuppression

Preclinical studies have shown that the immunosuppressive function of MDSCs is enhanced by surgery. Therefore, anti-MDSC-mediated immunosuppression may disrupt premetastatic niche development after surgery. TGF-β is a vital cytokine for establishing an immunosuppressive pre-metastatic environment by MDSCs. Autocrine and paracrine TGF-β effects on MDSCs survival and lineage commitment suggest that pharmacologic TGF-β signaling inhibition might favor antitumor immunity.^166^ The phosphodiesterase 5 (PDE 5) inhibitors, sildenafil and tadalafil, have been reported to inhibit MDSCs mediated immunosuppression.^167^–^169^ Enhanced recovery after surgery (ERAS), aimed at rapidly restoring immunity after surgery and reducing surgical stress, has been found to benefit patients undergoing extensive solid cancer surgeries. The ERAS pathway has been associated with effective postoperative analgesia, major reductions in in-hospital opioids consumption, and reduced pain, relative to conventional management practices.^170^ Moreover, preoperative multimodal exercise and nutritional programs (prehabilitation) are associated with improved 5-year disease free survival in stage III colorectal cancer after surgical resection.^171^ In colorectal cancer patients, application of the ERAS protocol is associated with low inflammatory metrics on postoperative day 1, and improved 5-year survival after surgery.^172^

## Conclusions and Perspectives

The roles of MDSCs in cancer progression have been well elucidated. However, more attention should be paid to the period immediately after surgery. During this period, the complex interplay between immune responses and dysfunction determines the long-term survival. The 2 weeks of MDSC persistence in lungs after surgery produces a premetastatic niche that makes the lungs more vulnerable to colonization by circulating cancer cells. More studies are needed to identify the phenotypes and function of postsurgical MDSCs given that they may be influenced by multiple factors that respond to surgical stress. Surgical stress initiates an inflammatory response that promotes MDSCs development and mobilization. In addition, surgical trauma releases DAMPs into circulation, which trigger local defense responses upon arrival at distant organs, including lungs. Local inflammatory responses to DAMPs lead to chemokine release, which attract MDSCs. DAMPs also directly activate MDSCs through the NF-κB and STAT3 signaling pathways. ECM remodeling and enhanced vascular permeability induced by surgery promote MDSC settlement, which enhances the development of a premetastatic microenvironment. Anti-postoperative MDSC infiltration therapies disrupt the premetastatic niche and improve long-term survival outcomes after tumor resection. Most potential therapies are currently in the preclinical evaluation phases. Suppression of surgical stress effects using COX-2 inhibitors or β-blockade have shown favorable effects in preclinical studies. Anti-DAMPs and associated inflammatory signals, such as NF-κB, IL-1β, and NLRP3 inflammasome, are rational therapies. Chemokine receptor inhibitors, in combination with low-dose epigenetic drugs have been shown to disrupt premetastatic niche formation, thereby improving long-term survival outcomes in mice. Inhibition of postoperative ECM remodeling and vascular leakage repair also could be beneficial during the perioperative period. Moreover, anti-MDSC therapies in tumor-bearing conditions, including low-dose chemotherapy, TKIs, and ATRA are promising therapeutic options in the perioperative period. However, the dosage and timing of these drugs, as well as the immunosuppressive status and associated complications, should be carefully considered. Finally, reversing the postoperative immunosuppressive state using TGF-β inhibitors or the ERAS protocol has the potential to improve the long-term survival outcomes of patients after tumor resection.
